# Incidence of HCV induced—Esophageal varices in Egypt: Valuable knowledge using data mining analysis

**DOI:** 10.1097/MD.0000000000005647

**Published:** 2017-01-27

**Authors:** Mahmoud Abdel-aty, Mahmoud Fouad, Mohammad M. Sallam, Elsayed A. Elgohary, Ali Ismael, Abdallah Nawara, Baha Hawary, Mohammed Tag-Adeen, Salama Khaled

**Affiliations:** a Department of Mathematics and Information Technology, Zewail City for Biosciences and Technology, Giza; b Department of Gynecology and Obstetrics, Al Azhar Asuit Faculty of Medicine, Al Azhar University; c Department of Internal Medicine, Zagazig Faculty of Medicine, Zagazig University; d Department of Pediatrics and Neonatology, Aswan School of Medicine, Aswan University; e Department of Internal Medicine, Qena Faculty of Meidicne, South Valley University; f Department of Gastroenetrology, Nagazaki School of medicine, Nagazaki University, Japan; g Department of Gastroenterology and Hepatology, Nasser Institute Hospital for Research and Therapy, Cairo, Egypt.

**Keywords:** cirrhosis, data mining, decision tree, Egypt, esophageal varices

## Abstract

Esophageal varices is one of the most important comorbidity related liver cirrhosis, patients usually presented with hematemesis, melena, or both, ultimately 20% is the mortality during the first attack, hence we aimed to investigate the incidence of such esophageal varices related chronic Hepatitis C virus (HCV) in randomized Egyptian population.

One thousand eighteen Egyptian patients, aged between 17 and 58 years, positive for Hepatitis C virus genotype 4 (HCV-4) by enzyme linked immunosorbent assay Ab and HCV RNA-polymerase chain reaction were screened for the presence of esophageal varices.

Incidence of esophageal varices was 62.3%; 635 patients, those with large Esophageal varices (LEVs) was 47.4%; 301 patients. Model for end-stage liver disease (MELD) score has not been significantly improved post variceal band ligation (VBL). Using 2D U/S was useful for EVs prediction.

Incidence of esophageal varices in HCV Egyptian patients still high, valuable knowledge would be helpful in clinical field have been discovered by data mining computational intelligent analysis using in practical medicine to improve overall health care.

## 1 Introduction

Hepatitis C virus (HCV) has been identified by the World Health Organization (WHO) as a major health problem; accordingly HCV is a major cause of chronic liver disease, hepatocellular carcinoma, and deaths from liver disease and is the most common indication for liver transplantation worldwide. Portal hypertension induced Esophageal varices is one of life threatening complication of liver cell failure, lead to much mortalities and comorbidities, additionally affect overall health preparation for liver transplantation.^[1–4]^ Recently data mining computational analysis can predict disease progression and regression in an intelligent fashion of decision tree and naïve Bayes analyses, would affect the overall health care systems, that the challenge for biomedical researchers is to make these theoretical analyses are much applicable in clinical medicine.^[5]^ Here, we will present useful applicable data about incidence of esophageal varices related HCV-4 in such randomized Egyptian population using the intelligent computational analyses.

## 2 Patients and methods

Between January 2004 and May 2012, 1018 patients (627 men and 391 women), positive for both HCV Ab enzyme linked immunosorbent assay (ELISA) third generation application and quantitative polymerase chain reaction (PCR) test.

All patients were HCV genotype 4, all of them presented in variable hepatitis degree from mild hepatitis activity to decompensated cirrhosis, many patients experienced failed Peg. Interferon-Ribavirin combined therapy, other did not experience the combined therapy due to contraindication, decompensation, or those denied therapy. Patients were positive to HCV Ab and negative by qualitative PCR test were excluded from the study.

Patients with esophageal varices were included in our internship program to evaluate esophageal variceal degrees using non-invasive 2D U/S.

## 3 Statistical analysis

Ten folds cross-validation using naive Bayes application. Sensitivity was 97%, specificity was 93% whatever the overall accuracy was 95%. A descriptive model was generated using a decision tree algorithm. The decision tree decided the most significant independent variable in each stage of predicting dependent variables. (Using the Rapid Miner, Rapid I, version 4.6, Berlin, Germany).

## 4 Results

One thousand eighteen Egyptian patients, infected with HCV, confirmed by both; ELISA; third generation assay and HCV-RNA PCR test, man affection is significantly higher than woman; 638; (62.7%) versus 380; (37.3%), in randomized Egyptian population group in both urban and rural areas.

Out of 1018 patients, only 635 patients 62.3%, (398 men; 62.7% and 237 women; 37.3%) aged between 17 and 58 years old, presented with cirrhotic-portal hypertension criteria, whatever 383 (37.62%) did not show any cirrhotic manifestations.

**Figure 1:**
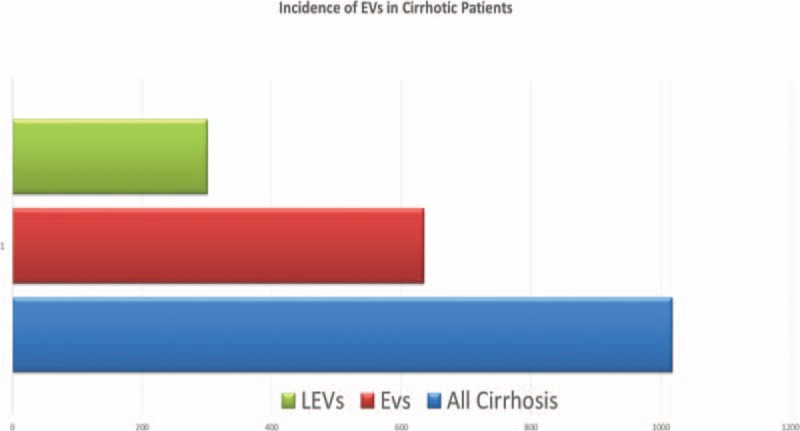
Charts of incidence esophageal variceal degrees.

In those presented with cirrhosis, only 301 (47.4%), 211 men (70%) and 90 women (30%), had large esophageal varices; Grade III and Grade IV varices (Table 1, Fig. 1).

**Table 1:**
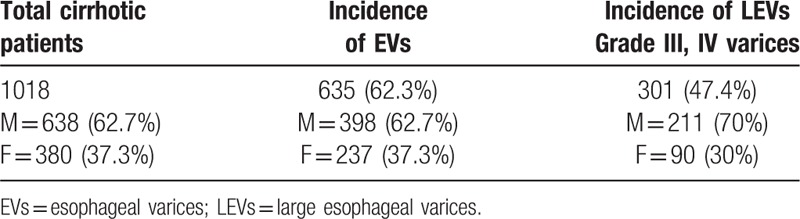
Showing incidence of esophageal varices in randomized Egyptian population group.

**Figure 2:**
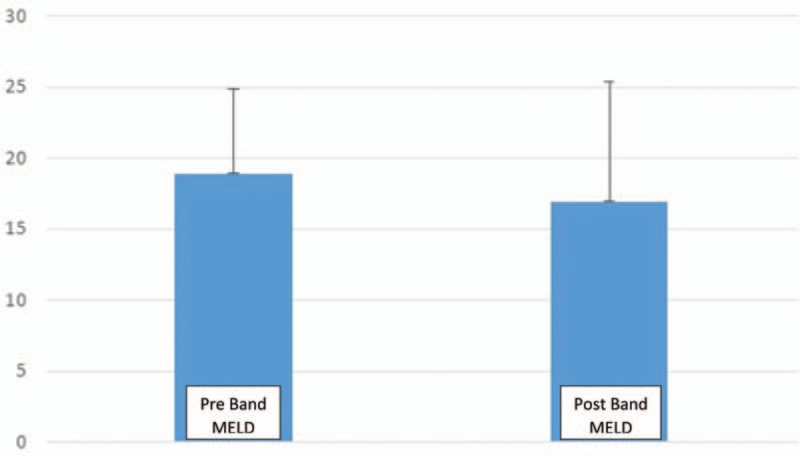
Charts pre and postband MELD score. MELD = model for end-stage liver disease.

Model for end-stage liver disease (MELD) score have not been improved significantly post band ligation (Table 2, Fig. 2).

**Table 2:**
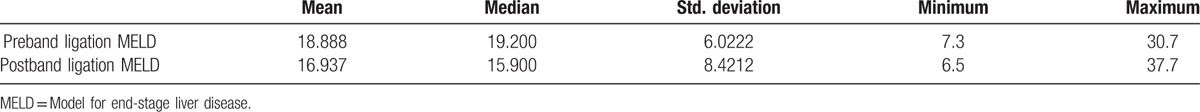
Showing MELD score pre and postband ligation *P* = 0.5 (not significant).

**Figure 3:**
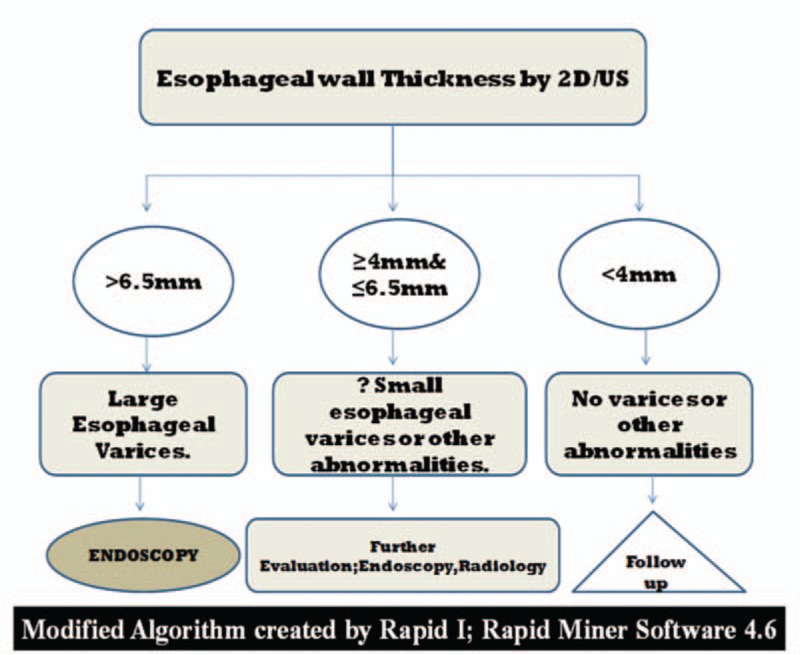
Algorithm created by Decision tree of RapidI Miner ver.4.6 showing incidence of esophageal variceal degrees for each esophageal wall thickness measured by conventional 2D US. US = ultrasound.

**Figure 4:**
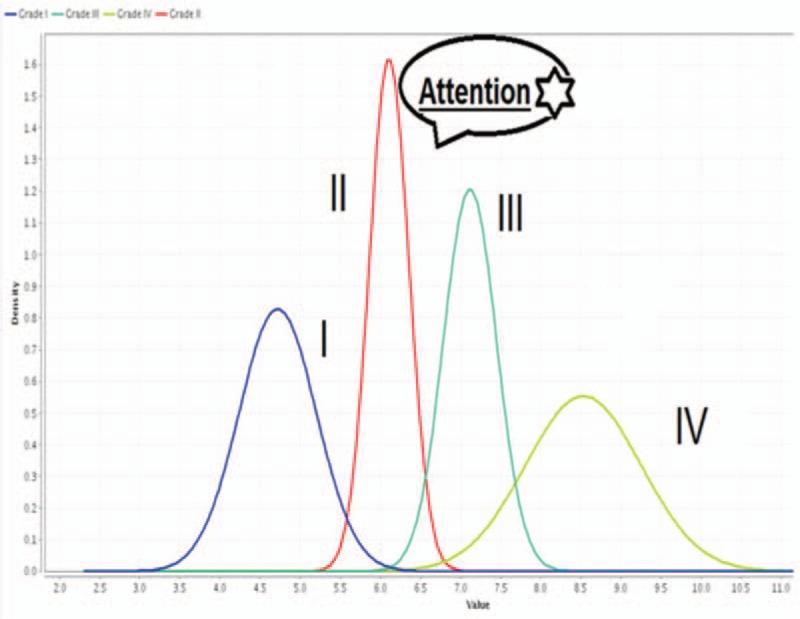
Naïve Bayes analyses of esophageal wall thicknesses corresponding to different esophageal varices. Data mining recommendation is to give prophylactic measures when esophageal wall thickness ≥6 mm.

**Figure 5:**
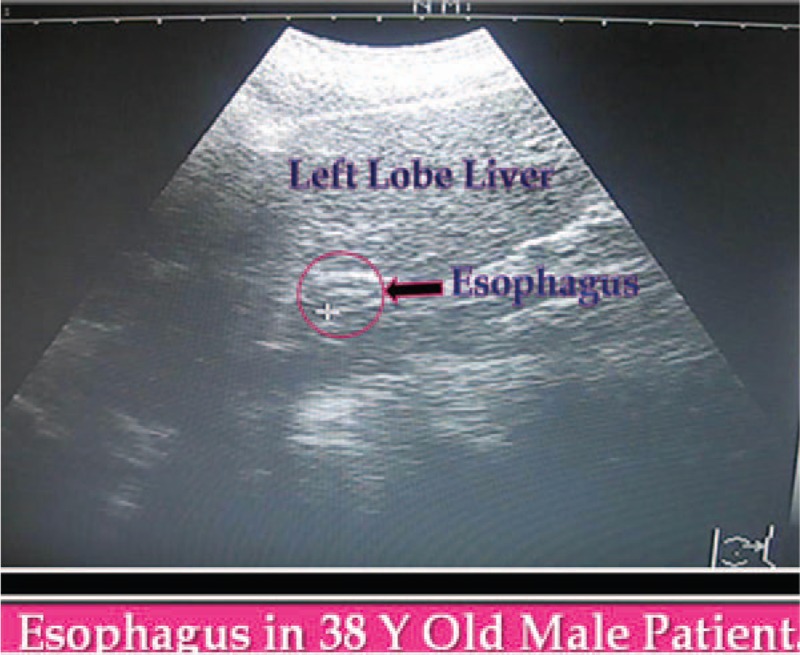
Conventional 2D US showing intra-abdominal portion of esophagus in male patient 38 years old. US = ultrasound.

Modified decision tree created by data mining showing that esophageal wall thicknesses >6.5 mm using 2D U/S were correlated with large esophageal varices; Grade III or IV varices, detected by upper endoscopy, whatever esophageal wall thicknesses less than 4 mm correlated with normal endoscopic finding; no varices or other abnormalities (Figs. 3–5).

## 5 Discussion

Globally, it was estimated that, Hepatitis C virus genotype 4 (HCV-4) is the cause of approximately 20% of the 180 million cases of chronic hepatitis C in the world. HCV-4 infection is common in the Middle East and Africa. HCV prevalence is highest in Egypt at >10% of the general population and China has the most people with HCV (29.8 million).^[6]^ However, after the evolution of direct acting antivirals (DAAs) there is a hope towards a safer world of HCV within coming 10 years, unfortunately DAAs will not treat liver cell failure or portal hypertension induced esophageal varices. Hence, the world may face mortality and comorbidity related HCV complications even for cured patients; positive sustained virologic response (SVR).

Upper gastrointestinal bleeding is a common medical condition that results in high patient morbidity and medical care costs, usually patients presenting with hematemesis (vomiting of blood) and/or melena (black, tarry stool) due to portal hypertension-induced cirrhosis. Esophago-gastro-duodenoscopy (EGD) is the diagnostic modality of choice for acute upper GI bleeding due to high sensitivity and specificity. Current study showed that the prevalence of HCV induced—cirrhosis is more common in men than women, provides an evidence in favor of a higher HCV clearance rate in women compared with men because of evident—suppression activity of estrogen against HCV replication in women, additionally progression of hepatitis C virus (HCV) infection is known to be worse in men than in women, independent of alcohol intake, men have a twofold greater progression rate to fibrosis compared with women.^[7–13]^

MELD score, is a scoring system for assessing the severity of chronic liver disease. It was initially developed to predict death within 3 months of surgery in patients who had undergone a trans-jugular intrahepatic portosystemic shunt (TIPS) procedure, and was subsequently found to be useful in determining prognosis and prioritizing for receipt of a liver transplant. This score has been using by the United Network for Organ Sharing (UNOS) and Euro-transplant for prioritizing allocation of liver transplants instead of the older Child-Pugh score.^[14,15]^ Those cirrhotic patients presented with large Esophageal varices (LEVs) prepared for liver transplantation should receive variceal band ligation (VBL) before surgery, hence we evaluated their MELD before and post VBL, it was no significant improvement in scoring MELD (Table 2, Fig. 3).

Recently, non-invasive 2D U/S can detect risky esophageal varices in cirrhotic patients. Furthermore, esophageal wall thicknesses less than 4 mm correlated with normal endoscopic finding; suggesting no varices. Screening for esophageal varices using such noninvasive technique, should be helpful in monitoring patients with liver cirrhosis.^[16]^

Data mining showed the association between esophageal wall thicknesses and corresponding variceal degrees in a proportional fashion; the median wall thicknesses for Grade I varices was 4.75 mm, for Grade II varices (6.25 mm), for Grade III varices (7.25 mm), and for Grade IV varices (8.50 mm). Additionally, it was interesting that giving prophylactic medications when esophageal wall thicknesses at 6 mm; Grade II varices (attention mark; Fig. 3) will significantly decrease small esophageal varices progression to Grade III and IV varices, would decrease overall mortality and morbidity Figs. 3–5.

Prophylactic band ligation with pharmacotherapy in the form of non-selective beta blocker (40 mg in 2 divided doses and Isosorbide-mononitrate 20–25 mg/daily) was very effective as primary and/or secondary prophylaxis in those presented with esophageal varices, however most of Egyptian patients cannot tolerate doses more than those mentioned above may need more explanation in future studies.

Patients presented with gastric varices should to be injected with Cyanoacrylate compounds. All women should receive antenatal screening for both HBsAg and HCV Ab. It is particularly interesting that in Egyptian rural villages the prevalence of anti-HCV reaches a mean of 15.5%, with a 30% rate in women aged >35 years. The overall rate of mother-to-child transmission of HCV from HCV-infected, HIV-negative mothers has been estimated at 3% to 5%. Coinfection with HIV increases the rate of mother-to-child transmission up to 19.4%. The detection of HCV RNA in the serum of infants in the first 24 hours of life suggests that early intrauterine infection may be possible. Numerous risk factors for vertical transmission have been studied. In general, high viral load defined as at least 3 × 10^6^ viral RNA copies/mL, HIV coinfection, and invasive procedures are the most important factors. Additionally, a Japanese study suggested that maternal liver dysfunction, large blood loss at delivery, and vaginal delivery were potential novel risk factors for mother-to-child transmission of HCV.^[17–23]^ Whatever pregnancy is not common on top of liver cirrhosis of chronic HCV infection, we experienced many women with esophageal varices and portal hypertension during pregnancy, pregnancy termination was done for those presented with massive attacks of hematemesis due to mother hypovolemic shock and bleeding tendency in a part and/or intra-uterine fetal death, hence evaluation of esophageal wall thickness by non-invasive U/S was very helpful non-invasive tool during pregnancy assessing esophageal variceal degrees. We recommend cesarean section with prophylactic somatostatin analogues in those with evident EV by ultrasound (US) at the time of delivery. Additionally, we reported many pediatric cases of gastro-esophageal varices of HCV chronic infection suggested early childhood or even intra-uterine transmission, mortality in such HCV infected children was high with much comorbidity of rebleeding attacks (non-published data), however conventional US was helpful to distinguish children presented with hematemesis due to esophageal varices from those presented due to other causes before EGD, furthermore we need guidelines recommendations of DAAs safety for such pediatric population in Egypt.

Data mining is the breakthrough in advanced medicine, holding the greatest potential for the healthcare industry to enable health systems to systematically use data and analytics to identify factors related diseases progression and regression and to qualify best practices that improve care and reduce costs significantly. We believe the opportunities to improve health care and reduce costs in Egypt may exceed as much as 40% of overall healthcare spending (study under completion). One of very applicable tool of data mining using in medicine the decision tree algorithm, trying to mimic the human brain connecting attributes to each other, aiming to compare these information-related attributes to one another, finally looking for the strongest connections. The network could apply the suitable required model to score the applicable data to make predictions in the applied medicine.^[24,25]^

## 6 Limitation of the study

The study was performed before initiation of DAAs, hence we suggest another study prevalence of Esophageal varies in Egyptian population after the evolution of DAAS.
